# Supplementary material for: Lipidomics-Based Comparison of Molecular Compositions of Green, Yellow, and Red Bell Peppers
Source: Metabolites. 2021 Apr 14;11(4):241. doi: 10.3390/metabo11040241 (PMC8070949; doi:10.3390/metabo11040241)
Supplement: Supplementary file 1 [file metabolites-11-00241-s001.zip › metabolites-1185348-supp/Supplemental Methods_Compound Annotation.pdf]

Supplemental Methods: Compound annotation

Processed data was reimported into MPP for annotation using Agilent MassHunter ID Browser B.08 (ID Browser) to search in-house and commercial databases. The in-house database comprises Human Metabolome Database (HMDB) 4.0 [1], Lipid Maps [2], National Institute of Science and Technology (NIST) [3], and 638 authentic standards with MS/MS data. Annotations were based on accurate mass, with a mass error cutoff of 10 ppm, isotope ratios and isotopic distribution whereby the predicted isotope distribution is compared to actual ion height and a score is generated. Scores ≥ 50 were considered putative annotations and correspond to a Metabolomics Standards Initiative (MSI) metabolite identification level two or three [4]. In metabolomics, MSI level two is defined as compounds that have been annotated based on physiochemical properties or through matching to MS/MS spectral libraries. MSI level one is the highest form of annotation and requires matching LC/MS/MS data to authentic reference standards. In addition, unmatched data for significant compounds was manually searched using FoodDB [5], PubChem [6], and KNApSAcK [7]. For compounds in which no annotation was possible, the molecular formula generator in ID Browser was used to estimate a metabolite chemical formula. All data and annotations were also manually reviewed.

To improve confidence in annotations, tandem MS analysis (MS/MS) was performed by targeting m/z and RT of compounds of interest in pepper samples. Lipid extracts were run using the same LC-MS method as original samples (see main text for details) with MS/MS data collected at fixed 10, 20, and 40eV collision energies. Resulting experimental MS/MS spectra were compared in National Institute of Science and Technology (NIST) Tandem Mass Spectral Library (Version 2.3) [8, 9] using the NIST14 and NIST17 MSMS spectral libraries. One molecule of interest was initially annotated as β-cryptoxanthin following searching of commercial and in-house databases. Subsequent tandem mass spectrometry of pepper samples for the molecule confirmed this annotation with a 552 m/z and

matching to the NIST17 standard mass spectra library; match factor value for β-cryptoxanthin was 762 and the reverse match factor value was 887 (Supplementary Methods Figure 1).

Supplemental Figure 1A.

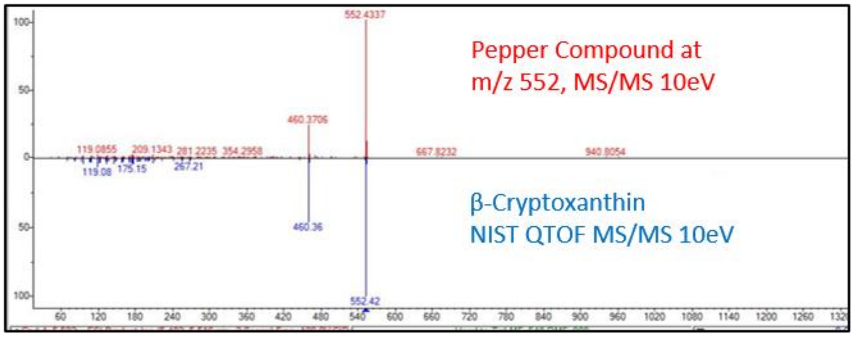

Supplemental Figure 1B.

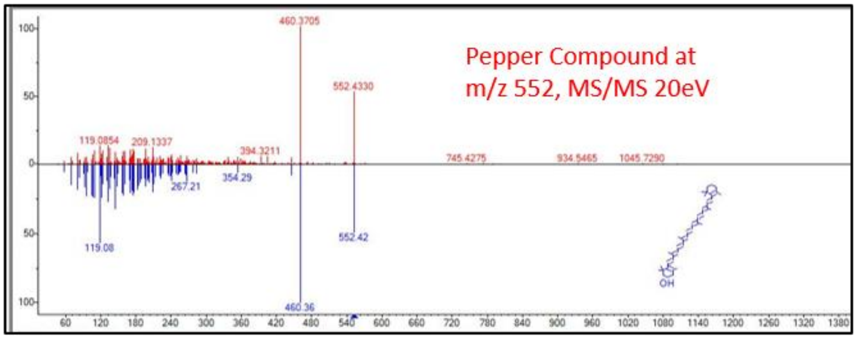

Supplemental Figure 1C.

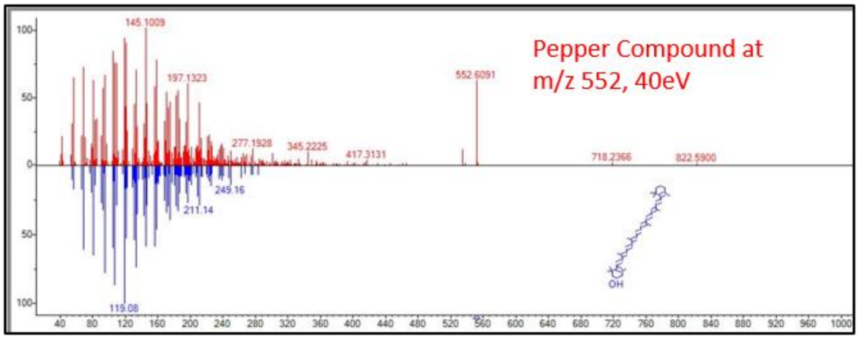

Supplemental Methods Figure 1: MS/MS of compound initially annotated as β-cryptoxanthin. Figure shows NIST17 MS/MS Difference results spectra for pepper compound at m/z 552 using collision energies at 10eV (S1A), 20eV(S1B), and 40eV(S1C) matched to β-cryptoxanthin from the NIST QTOF spectral library. The top spectra (red) is the experimental pepper compound MS/MS spectra, the bottom spectra (blue) is from the NIST QTOF spectral fragments.

While this confirmed the annotation, additional review showed that β-cryptoxanthin is indistinguishable from α-cryptoxanthin, both of which are 552 m/z and have identical MS/MS spectra. Authentic standards of β-cryptoxanthin and α-cryptoxanthin were purchased, analyzed using liquid chromatography along with drift tube ion mobility mass spectrometry (Agilent 6560 IMMS-QTOF, Santa Clara, CA) to determine if the α and β isoforms could be resolved. While the α and β isoforms had slightly different retention times, they had the same drift time (Supplemental Methods Figure 2). These results are generally consistent with previous work [10], whereby separation of α and β isoforms was achieved using a different HPLC method then was employed in the current study. Because α-cryptoxanthin is rarely found in plants [10], it seemed most plausible that β-cryptoxanthin was present in peppers and we thus considered this the most likely annotation.

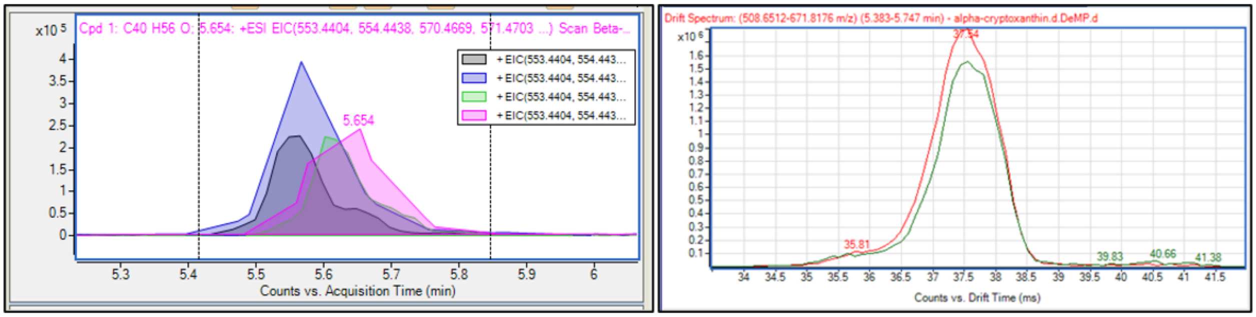

Supplemental Methods Figure 2: **Analysis of cryptoxanthin standards using ion mobility mass spectrometry. Left.** Extracted Ion Chromatogram (EIC) showing the α form of cryptoxanthin in Purple and Gray, and the β form in Green and Pink. **Right**. Drift Spectrum showing the α form in Red and the β form in Black.

Tandem MS/MS data was also acquired for remaining compounds of interest (Table 1, Main Text); however, usable data was only obtained for 4 additional compounds. All MS/MS data was searched using the NIST17 spectral libraries but no spectral matches were observed. The data was then searched using the in silico MS/MS spectral interpretation software SIRIUS version 4.6.0 [11] for formula and CSI:FingerID version 1.4.8 [12] for compound annotation.

To increase confidence in compound ID database searches, pepper compounds were limited to the following natural product databases: Collection of Open Natural Products (COCONUT) [13], Global Natural Products Social Molecular Networking (GNPS) [14] , Plant Metabolic Network (PMN) [15], KNApSAcK [7], and SUPER NATURAL II [16].

In these cases, the MS/MS data did not match the original database search results and additional interpretation was required. Annotations in the main text indicate the best matches following MS, MS/MS, in silico searches and overall likelihood of a compound being present in plants/peppers. Alternate names are listed in Supplemental Methods Tables 1 and 2.

Supplemental Methods Table 1: Alternate compound names based on MS and/or tandem MS

Reported Annotation	Reported based on	Alternative Annotations
Beta-cryptoxanthin	MS and MS/MS	Alpha-cryptoxanthin
2-ethenyl-2,4b,8,8-tetramethyl-tetradecahydrophenanthrene-3,5,10a-triol	MS/MS	Original annotation was PGF2
Fargesin	MS and likelihood of presence in sample	Lansiumarin B
Ascorbyl linoleate	MS and likelihood of presence in sample	Fusicoplagin A, Agaric acid
Sucrose acetate isobutyrate	MS	No alternative hits
Ivermectin B1b	MS	No alternative hits
Glycidyl oleate	MS	No alternative hits
Goyaglycoside g	MS	No alternative hits
Ramipril	MS	No alternative hits
all-trans-retinyl oleate	MS	No alternative hits
Archaetidylglycerol-myo-inositol	MS	No alternative hits

Supplemental Methods Table 2: Alternate compound names based on in silico analysis

Original MPP Annotations						SIRIUS RESULTS								
**Compound**	**Mass**	**MS1 Composite Spectrum**	**Ion Species**	**Retention Time**	**Formula**	**Name**	**Formula**	**m/z**	**ppm Error**	**Adduct**	**# Explained Peaks**	**Tree Score**	**Fragment TIC**	**Database Sources**
PGF2alpha-d4	318.2748	(**341.2646**, 8505.0)(342.264, 1318.15)	[M+Na]+	1.324	C20 H30 D4 O5	2-ethenyl-2,4b,8,8-tetramethyl-tetradecahydrophenanthrene-3,5,10a-triol	C20 H34 O3	341.2687	4.33	M+H-H2O	10/30	41.89	28.62%	COCONUT, SuperNatural II
Fusicoplagin A	438.2597	(**439.27**, 116694.81)(440.2731, 28633.93)(441.2783, 6081.88)	[M+H]+	7.403	C24 H38 O7	Agaric acid	C22 H40 O7	439.2682	3.54	M+Na	30/15	88.18	75.53%	COCONUT, SuperNatural II

References

1. Wishart, D.S., et al., *HMDB 4.0: the human metabolome database for 2018.* Nucleic Acids Res, 2018. **46**(D1): p. D608-D617.
2. O'Donnell, V.B., et al., *LIPID MAPS: Serving the next generation of lipid researchers with tools, resources, data, and training.* Sci Signal, 2019. **12**(563).
3. Choquette, S.J., D.L. Duewer, and K.E. Sharpless, *NIST Reference Materials: Utility and Future.* Annu Rev Anal Chem (Palo Alto Calif), 2020. **13**(1): p. 453-474.
4. Sumner, L.W., et al., *Proposed minimum reporting standards for chemical analysis Chemical Analysis Working Group (CAWG) Metabolomics Standards Initiative (MSI).* Metabolomics, 2007. **3**(3): p. 211-221.
5. Harrington, R.A., et al., *Nutrient composition databases in the age of big data: foodDB, a comprehensive, real-time database infrastructure.* BMJ Open, 2019. **9**(6): p. e026652.
6. Wang, Y., et al., *PubChem BioAssay: 2017 update.* Nucleic Acids Res, 2017. **45**(D1): p. D955-D963.
7. Afendi, F.M., et al., *KNApSAcK family databases: integrated metabolite-plant species databases for multifaceted plant research.* Plant Cell Physiol, 2012. **53**(2): p. e1.
8. Stein, S.E., *Estimating probabilities of correct identification from results of mass spectral library searches.* J Am Soc Mass Spectrom, 1994. **5**(4): p. 316-23.
9. Technology, N.I.o.S.a. *NIH Mass Spectral Library with Seach Program (Data Verison: NIST 14, Software Version 2.2)*. 2014; Available from: http://www.nist.gov/srd/nist1a.cfm.
10. de Azevedo-Meleiro, C.H. and D.B. Rodriguez-Amaya, *Qualitative and quantitative differences in the carotenoid composition of yellow and red peppers determined by HPLC-DAD-MS.* J Sep Sci, 2009. **32**(21): p. 3652-8.
11. Duhrkop, K., et al., *SIRIUS 4: a rapid tool for turning tandem mass spectra into metabolite structure information.* Nat Methods, 2019. **16**(4): p. 299-302.
12. Duhrkop, K., et al., *Searching molecular structure databases with tandem mass spectra using CSI:FingerID.* Proc Natl Acad Sci U S A, 2015. **112**(41): p. 12580-5.
13. Sorokina, M., et al., *COCONUT online: Collection of Open Natural Products database.* J Cheminform, 2021. **13**(1): p. 2.
14. Wang, M., et al., *Sharing and community curation of mass spectrometry data with Global Natural Products Social Molecular Networking.* Nat Biotechnol, 2016. **34**(8): p. 828-837.
15. Dreher, K., *Putting The Plant Metabolic Network pathway databases to work: going offline to gain new capabilities.* Methods Mol Biol, 2014. **1083**: p. 151-71.
16. Banerjee, P., et al., *Super Natural II--a database of natural products.* Nucleic Acids Res, 2015. **43**(Database issue): p. D935-9.
